# Soil Landscape Pattern Changes in Response to Rural Anthropogenic Activity across Tiaoxi Watershed, China

**DOI:** 10.1371/journal.pone.0166224

**Published:** 2016-11-10

**Authors:** Rui Xiao, Diwei Jiang, George Christakos, Xufeng Fei, Jiaping Wu

**Affiliations:** 1 Institute of Islands and Coastal Ecosystems, Zhejiang University, Zhoushan, China; 2 School of Remote Sensing and Information Engineering, Wuhan University, Wuhan, China; Pacific Northwest National Laboratory, UNITED STATES

## Abstract

Soil sealing (loss of soil resources due to extensive land covering for the purpose of house building, road construction etc.) and subsequent soil landscape pattern changes constitute typical environmental problems in many places worldwide. Previous studies concentrated on soil sealing in urbanized regions, whereas rural areas have not been given sufficient attention. Accordingly, this paper studies soil landscape pattern dynamics (i.e., landscape pattern changes in response to rural anthropogenic activities) in the Tiaoxi watershed (Zhejiang province, eastern China), in which surface sealing is by far the predominant component of human forcing with respect to environmental change. A novel approach of quantifying the impacts of rural anthropogenic activities on soil resources is presented. Specifically, quantitative relationships were derived between five soil landscape pattern metrics (patch density, edge density, shape index, Shannon’s diversity index and aggregation index) and three rural anthropogenic activity indicators (anthropogenic activity intensity, distance to towns, and distance to roads) at two landscape block scales (3 and 5 km) between 1985 and 2010. The results showed that the Tiaoxi watershed experienced extensive rural settlement expansion and high rates of soil sealing. Soil landscapes became more fragmented, more irregular, more isolated, and less diverse. Relationships between soil landscape pattern changes and rural anthropogenic activities differed with the scale (spatial and temporal) and variable considered. In particular, the anthropogenic activity intensity was found to be the most important indicator explaining social development intensity, whereas the other two proximity indicators had a significant impact at certain temporal interval. In combination with scale effects, spatial dependency (correlation) was shown to play a key role that should be carefully taken into consideration in any relevant environmental study. Overall, the findings of this work suggest that soil sealing can be a critical human forcing issue with considerable consequences deserving serious attention by the experts, the public and the government alike.

## Introduction

The physical growth of urban areas in response to socio-economic development, commonly termed “urbanization”, is one of the most significant processes influencing local, regional and global environments [1, 2]. Following the open-door policy of 1978, accelerated urbanization has taken place across China, especially in the southeastern coastal areas [3, 4]. The environmental problems associated with urbanization were extremely distinct in large cities and the surrounding peri-urban areas experiencing intense anthropogenic activities [5]. However, rural anthropogenic activities (usually in the form of settlement expansion) have also aggressively intensified in recent past, posing a great threat to natural resources and causing a variety of environmental problems [6].

Soils are very important natural resources, since they provide the majority of food, livestock feed, fiber and biotic fuel, and also serve as a source of greenhouse gases and an integrated part of biogeochemical cycles [7]. Evidence shows that soil resources are under increasing pressure from rural anthropogenic activities. One of the most visible problems associated with rural settlement expansion is the surface sealing of soils. Soil surface sealing refers to the “loss of soil resources due to the covering of land for housing, roads or other construction work” [8], and it is a common result of rural anthropogenic activity and infrastructure construction [9].

Soil sealing is generally a permanent process that exerts adverse effects on environmental quality, ecosystem services [10], urban climate and runoff [11]. Soil landscape refers to the spatial distribution of soil cover [12], which exhibits the full array of attributes describing soil type, soil properties, and associated landscapes. Soil sealing can change the mosaic patterns of soil landscapes. Yet, research concerning soil sealing and soil landscape pattern change in response to rural anthropogenic activities is very limited.

Rural human settlements exhibit different characteristics at different spatial scales. Soil landscape patterns respond rather differently to changing rural human settlements reflecting the multiple-scale structure of the landscape pattern. Therefore, analyzing anthropogenic activity effects on soil landscape patterns at different scales can provide a comparative evaluation of case studies in different areas, during different time periods, and at different observation scales. Interestingly, previous research rarely considered this kind of effects when studying soil landscape pattern changes and the influence of anthropogenic activities.

This work focuses on soil surface sealing, because in recent years this kind of sealing is among the most important factors of human forcing in China (this is also the case of the Tiaoxi watershed considered in this work, which is an important watershed in eastern coastal China). Moreover, the Tiaoxi surface sealing situation is applicable in many developed regions of China, which means that the proposed approach may be relevant and generalizable to other real-world situations in China. In view of these considerations, the objectives of this paper are to (1) identify soil sealing patterns due to rural anthropogenic activity in the Tiaoxi watershed; (2) investigate soil landscape pattern changes and their spatial variations during three different time periods (1985–1994, 1994–2003 and 2003–2010); and (3) derive and interpret quantitative relationships between soil landscape patterns and rural anthropogenic activity at different spatial scales.

### Study area

**Fig 1:**
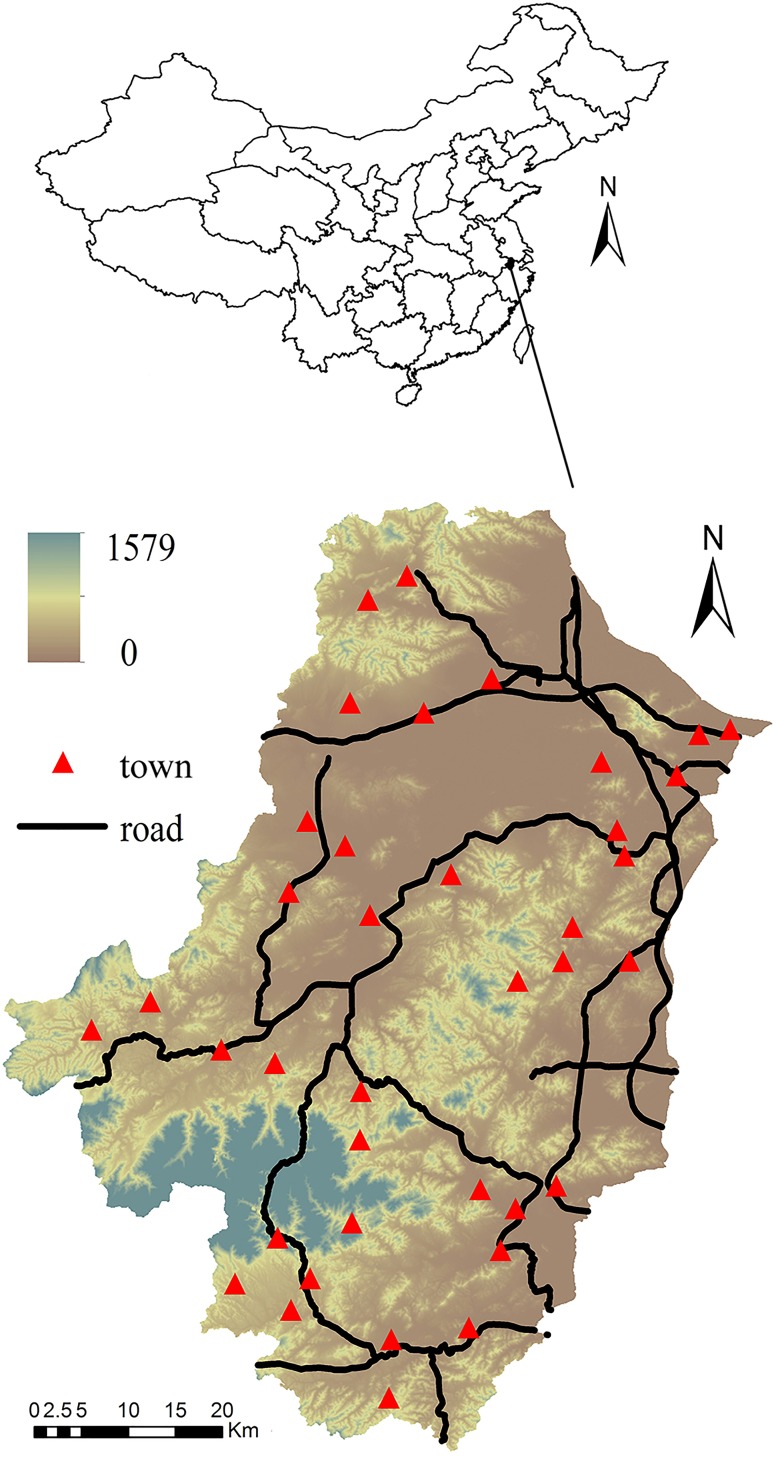
Location, spatial distribution of towns and major roads of the Tiaoxi watershed, China (legend indicates elevation above sea level, in meters).

**Fig 2:**
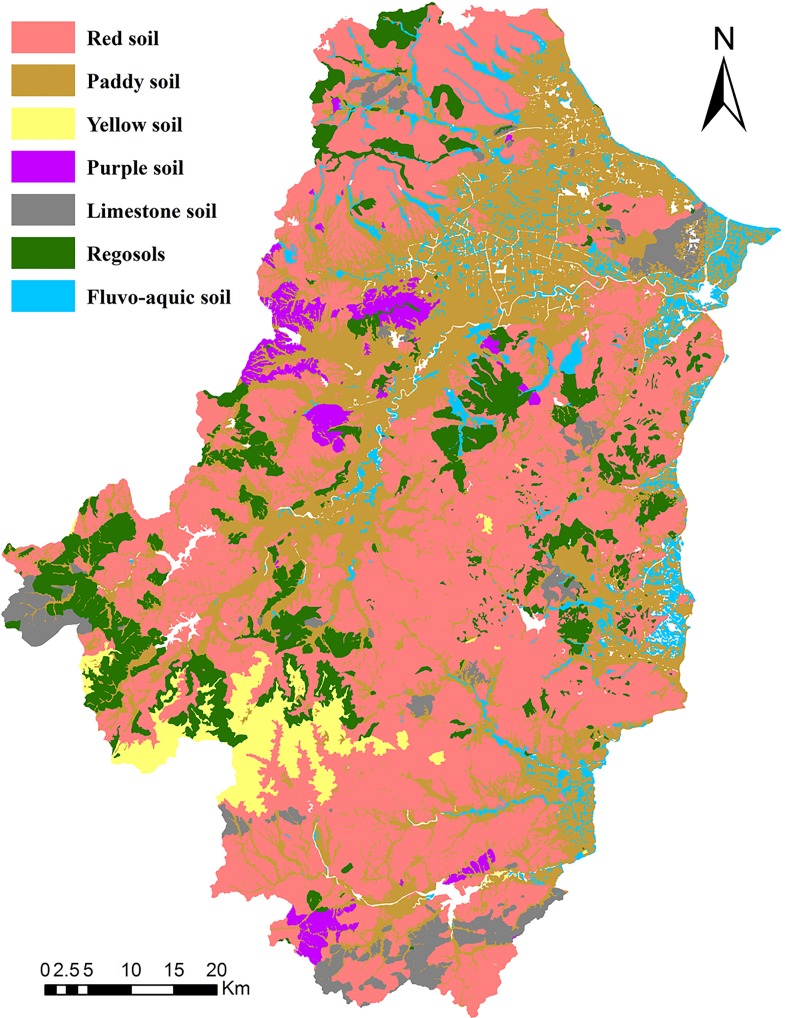
Soil Group map of the Tiaoxi watershed.

The Tiaoxi watershed, ranging from 30°07N’ to 31°11’N and from 119°14’ to 120°13’E, is located in the northern part of Zhejiang Province, eastern coast of China (Fig 1). It covers 6,000 km^2^ and has 4.3 million residents. This region has an average annual temperature of 17.5°C and rainfall of 1500 mm, with a subtropical monsoon climate. Red soil is the dominant soil type, accounting for 47.06% of the total area. The second dominant soil type is paddy soil, accounting for 28.31% of the total area. Other soil types include yellow soil (3.29%), purple soil (2.31%), limestone soil (3.97%), regosols soil (10.31%), and fluvo-aquic soil (4.76%) (Fig 2) [13]. The region’s topography is slanted downward from south west to east/northeast, and the mountain heights decrease from 1500 meters to merely 3–5 meters above the sea level from southwest to northeast.

Starting in 1978, when China adopted its open-door policy, rapid socio-economic development and population growth occurred in the Tiaoxi watershed. The Gross Domestic Product (GDP) was less than 3 billion RMB in 1985, but exceeded the 150 billion RMB in 2010. In addition, its population density amounted to 438 and 478 persons per km2 in 1985 and in 2010, respectively. Remarkably, as has been documented in the relevant literature [6], rapid socio-economic development and a lack of land planning led to large-scale rural settlement expansion, which exerted significantly negative impacts on soil resources. In view of these facts, the Tiaoxi watershed is a typical example as regards the characterization of the impacts of rural anthropogenic activities on soil resources in modern China.

## Materials and Method

### Data and processing tools

**Fig 3:**
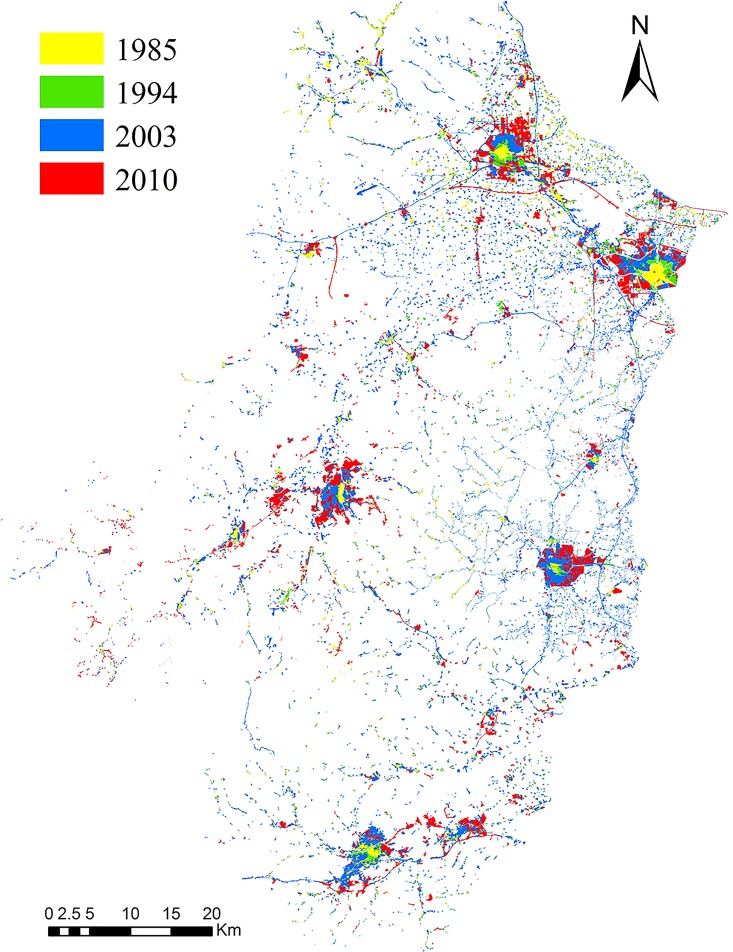
Spatial patterns of human settlements between 1985 and 2010 across Tiaoxi watershed.

Regional built-ups during the years 1985, 1994, 2003 and 2010 were obtained, using visual interpretation, from Landsat Thematic Mapper (TM) images (Path 119, Row 38–39; NASA and the U.S. Geological Survey) geo-referenced to UTM Zone 50 WGS 1984 coordinates [14]. Overlay analysis (facilitated by geographic information systems, GIS) was employed to delineate the expansion of rural settlements during the study period (Fig 3). The 2003 satellite data served as the basic image of visualization analysis and design, whereas on-screen enhancements were used in image interpretation. The built-up map prepared for the year 2003 was overlaid on the 1994 satellite image, and subsequently the 1994 built-up map was prepared. The vector maps of the 1985 and 2008 built-ups were prepared in a similar manner [15, 16]. The expanded built-up lands were first overlaid with the digital soil map (Fig 2). Then, the area of different soil types sealed by anthropogenic activity throughout the watershed was mapped.

### Metrics of soil landscape pattern

A wide variety of landscape metrics have been developed, which can be categorized into: (*a*) area/density/edge, (*b*) shape, (*c*) contagion/interspersion, and (*d*) diversity [17, 18]. The efficiency of the landscape metrics used in the present work to describe soil landscape patterns has been demonstrated in previous studies [14, 19]. In view of these studies, and in order to minimize redundancy among landscape metrics, we selected five landscape-level metrics from the above four categories: patch density (PD), edge density (ED), area-weighted mean shape index (SHAPE_AM), aggregation index (AI), and Shannon’s diversity index (SHDI) (Table 1).

**Table 1:** Landscape metrics.

Landscape metrics	Abbreviation	Description
Patch Density	PD	Number of patches per 100 ha.PD describes fragmentation.
Edge Density	ED	Total length of all edge segments per hectare. ED presents stability.
Area-Weighted Mean Shape Index	SHAPE_AM	Increase of SHAPE_AM means the landscape shapes became more complicated and irregular.
Aggregation index	AI	Aggregation index accounts only for the like adjacencies involving focal class, not adjacencies with other patch types. AI denotes aggregation.
Shannon’s diversity index	SHDI	SHDI refers to a quantitative measure that reflects how many different types there are in a dataset. SHDI denotes diversity.

We calculated the landscape metrics for soil mapping using Patch Analyst 4.0 [20] and AML scripts in ArcGIS 9.3 (ESRI Inc.). In order to identify the effect of scale on the relationships between soil landscape patterns and rural anthropogenic activity, a test was conducted using landscape block sizes ranging from 1 km to 20 km. After masking by built-up areas, soil information for each year was intersected with sampling blocks to incorporate soil property data into each block. The test indicated that 3 km and 5 km blocks were the best choices since they can retain enough soil information and adequately display the spatial variation of soil landscape patterns [19].

### Indicators of anthropogenic activity intensity

The anthropogenic activity intensity (*AAI*) indicator provides the means for ranking the relative intensity of rural development from low to high [21, 22]. This indicator can effectively capture the spatiotemporal dynamics of built-up land [19]. We used the same sampling blocks as in metric analysis above (3 km * 3 km and 5 km * 5 km) to intersect with the temporal land use maps and group the input data into each block. The AAI indicator is given by

$$AAI_{i}=\frac{A_{i,t+n}-A_{i,t}}{nW\delta_{i,t}}$$ (1)

Where *AAI*_*i*_ denotes anthropogenic activity intensity between year *t* and year *t+n* in block *i*; *A*_*i*,*t+n*_ and *A*_*i*,*t*_ represent the built-uplands in rural area in year *t+n* and year *t*, respectively; *n* is the temporal interval; *Wδ*_*i*,*t*_ is the total area of block *i* (sampling blocks for metric analysis were used as units of analysis). Therefore, *AAI*_*i*_ can range from -1 (100% negative development) to 0 (no change) to 1 (100% development).

Distance to towns (*Dis_t*) and distance to roads (*Dis_r*) indicators, which are associated with the intensity of anthropogenic activity and changes of landscape characteristics, can represent (and influence) the degree of built-up area expansion [19]. Notice that Dis_t and Dis_r have been widely used to assess the effects of anthropogenic activities on landscapes [23]. These facts justify our choice to employ the AAI, Dis_t, and Dis_r indicators to assess rural anthropogenic activity. In this context, it should be noticed that roads were under construction during the 25 years, however, for each time interval considered only the unchanged roads were used for analysis purposes.

### Spatial regression and anthropogenic activity-landscape pattern associations

Spatial regression was used to determine the relationships (associations) between soil landscape patterns (five metrics, in particular) and anthropogenic activity intensity (three indicators, above), instead of the simple univariate statistical techniques. This is a valid approach, since it has been documented in the relevant literature, the latter techniques may cause severe under-estimation [24].

In the present work, the space-time changes (dynamics) of landscape metrics were calculated using the following formula,

$$C_{i}=\frac{R_{2i}-R_{1i}}{R_{1i}}$$ (2)

where *C*_*i*_ denotes the change of landscape metric in block *i*; *R*_*1i*_ is the value of landscape metric in the preceding year; and *R*_*2i*_ is the value of landscape metrics in the following year.

Spatial regression models were employed to incorporate spatial dependence in the form of, (*a*) spatial lag models (in which the values of the dependent variables at adjacent sites exert a direct effect on the value of the dependent variable itself), and (*b*) spatial error models (in which spatial dependence enters through the error term rather than through the systematic model component). The spatial lag model used in the present analysis is given by [25],

$$Y_{i}=\rho \sum_{j}W_{ij}Y_{j}+X_{i}\beta +\varepsilon_{i},$$ (3)

where the subscript *i* represents spatial units at different scales; *Y*_*i*_ and *X*_*i*_ denote observations of, respectively, dependent variables (i.e., rural anthropogenic activity metrics, PD, ED, SHAPE_AM, SHDI and AI) and explanatory variables (i.e., soil landscape indicators, AAI, Dis_t, and Dis_r); *W*_*ij*_ are spatial weights, and *ε*_*i*_ are error terms; and *ρ*, *β* are model parameters. Also, the spatial error model is given by [25]

$$Y_{i}=X_{i}b+e_{i},$$ (4)

$$\text{with}\;e_{i}=\lambda \sum_{j}\omega_{ij}e_{j}+\mu_{i},$$ (5)

where *ω*_*ij*_ are spatial weights, ei are spatially correlated error terms, *μ*_*i*_ are uncorrelated error terms; and *λ*, *b* are model parameters (for technical details see [25], and for numerical values in the case of the Tiaoxi watershed see Table 2). All spatial regression models were computationally implemented using the GeoDa 0.9.5-i (Beta) software [26].

**Table 2:** Statistics of soil landscape metrics between 1985 and 2010 in the Tiaoxi watershed, China ^a^.

	1985	1994	2003	2010	Change rate (%) ^*b*^	Change rate (%)	Change rate (%)	Change rate (%)
					1985–1994	1994–2003	2003–2010	1985–2010
PD	0.86	0.87	1.13	1.22	1.85	29.66	8.22	42.91
ED	26.53	26.65	29.38	29.99	0.45	10.23	2.08	13.03
SHAPE_AM	15.23	15.30	14.62	12.67	0.41	-4.40	-13.35	-16.82
SHDI	0.17	0.17	0.15	0.14	-0.12	-11.43	-5.80	-16.67
AI	0.68	0.68	0.68	0.67	-0.10	-0.57	-0.41	-1.09

^*a*^Abbreviations: patch density (PD), edge density (ED), area-weighted shape index (SHAPE_AM), Shannon’s diversity index(SHDI), aggregation index (AI).

^*b*^Equations for calculation: Change rate = (R2-R1)/R1×100%, where R_1_ is the value at start year, R_2_ is the value at the end year.

## Results

### Patterns of soil sealing associated with settlement expansion

A considerable amount of soil was sealed by rural settlement expansion in the region of interest. Naturally, the sealed soil areas differed depending on the soil type (Table 3), with paddy soil being one of the most vulnerable types to be sealed. It was found that from 1994 to 2003, the area of sealed paddy soil was more than 14,000 ha (accounting for 2.5% of the total soil area), whereas more than 8,000 ha (accounting for 1.5% of the total soil area) were sealed during the period 2003–2010. Red soil and fluvo-aquic soil were substantially sealed between 1994 and 2003, with sealed areas exceeding 6,000 ha (accounting for about 1.1% of the total soil area) and 3,000 ha (accounting for 0.6% of the total soil area), respectively.

**Table 3:** Area of sealed soils during different temporal intervals in the Tiaoxi watershed (Unit: ha).

*Soil type*	*Different temporal intervals*		
	1985–1994	1994–2003	2003–2010
Paddy soil	1097.55	14206.86	8693.64
Red soil	194.94	6185.07	2802.78
Purple soil	7.74	148.05	218.16
Regosols	8.1	673.2	250.56
Limestone soil	173.16	868.5	467.46
Yellow soil	0.18	106.02	21.96
Fluvo-aquic soil	403.74	3233.07	1271.07

### Spatiotemporal changes of soil landscape patterns

Considering the temporal changes during the 25-year study period (1985–2010), which are represented by the metric values in Table 2, the soil landscapes in the Tiaoxi watershed became more fragmented (represented by increased PD and ED metric values with time averaged over the entire region), more irregularly shaped (decreased SHAPE metric values), more isolated (decreased AI metric values), and less diverse (decreased SHDI metric values). By comparing the temporal changes of landscape metrics (Table 2), it was found that these metrics experienced little change during the period 1985–1994 (less than 1.0%), but most of them exhibited high values during the period 1994–2003. Specifically, the change rate of PD during this period was 29.7%, whereas the ED and SHDI showed higher than 10% change rates. PD and ED exhibited increasing trends during the last 25 years and reached their peak in 2010. The other metrics generally showed a decreasing trend during the last 25 years and attained their lowest values in 2010. Comparing the period of 1985–1994 with that of 2003–2010, the latter time period experienced the highest rate of change.

**Fig 4:**
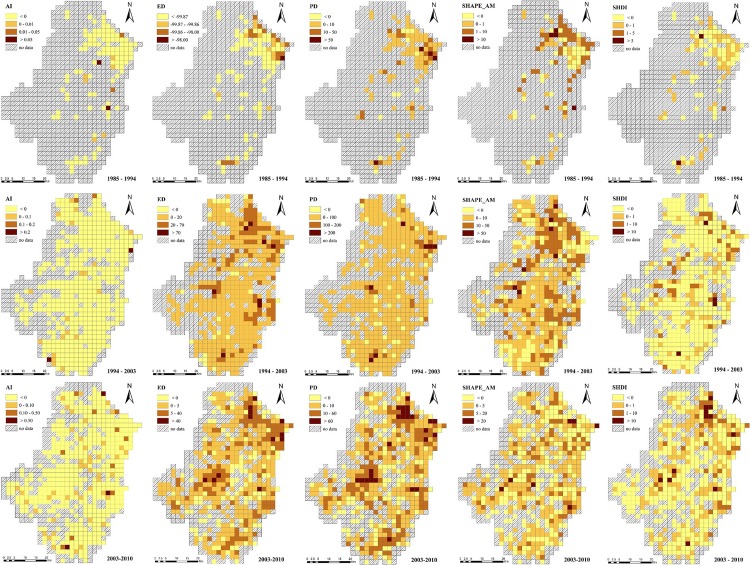
Spatiotemporal changes of soil landscape patterns at the 3 km scale between 1985 and 2010 across the Tiaoxi watershed.

**Fig 5:**
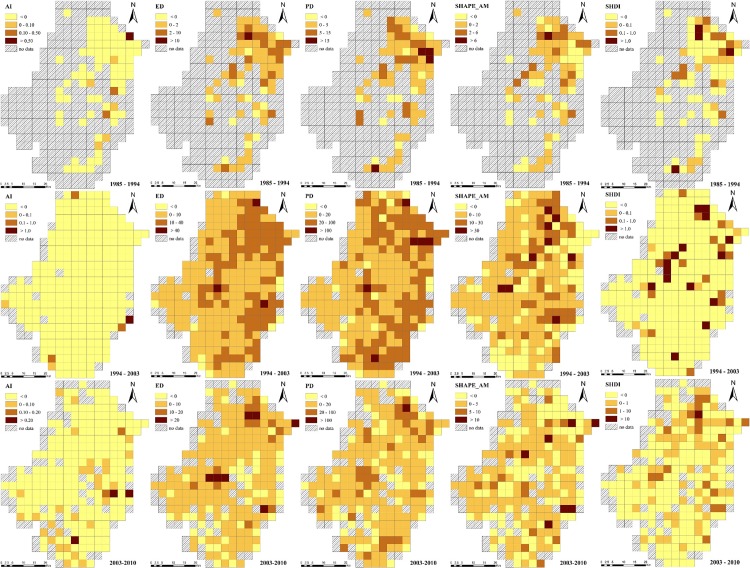
Spatiotemporal changes of soil landscape patterns at the 5 km scale between 1985 and 2010 across the Tiaoxi watershed.

As regards spatiotemporal changes of soil landscape. These changes varied significantly between 1985 and 2010 across the Tiaoxi watershed at the 3 km and 5 km scales (Fig 4 and Fig 5). More significant changes of landscape metrics were observed during the time period 1994–2003 than during the other two periods. The changes during the period 1985–1994 concentrated in the northeastern region, whereas during the period 1994–2003 the changes were evenly spread throughout the entire watershed (with a higher rate of expansion than during the period 1985–1994). Especially for the PD, ED and SHAPE metrics, the change was more significant not only in the northeastern region but also in the southeastern region. On the other hand, the AI and SHDI metrics showed decreasing trends in most areas at the study time-scale.

### Relationships between soil landscape patterns and rural anthropogenic activity

The quantitative relationships between soil landscape pattern changes and rural anthropogenic activity are presented in Table 4 (3km scale) and Table 5 (5km scale). Comparing the Dis_r values for different soil landscape metric changes, it was found that this anthropogenic activity indicator showed a negative correlation with the PD, ED, SHAPE, SHDI soil landscape pattern metrics, but a positive correlation with the AI metric, at both scales considered. Moreover, the AAI indicator was positively correlated with the PD, ED and SHDI metrics, but negatively correlated with the SHAPE and AI metrics, at both scales. On the other hand, the Dis_t indicator showed a positive correlation with the PD, ED and SHAPE metrics, whereas it experienced a negative correlation with the AI metric at the 3 km scale. At the 5 km scale, the Dis_t had a negative impact on PD, ED, AI and SHDI, whereas if exhibited a positive correlation with SHAPE.

**Table 4:** Relationships between soil landscape change and rural anthropogenic activity (3*km* scale) obtained by spatial regression.

*Dependent variable*(*Metric*)	*Independent variable* (*Indicator*)	*Time period*	*Spatial regression*	*R*^2^
PD	AAI	1985–1994	*Y* = 0.04*X* + 0.12*W* + 0.03^*a*^	.49**
		1994–2003	*Y* = 0.06*X* + 0.17*W* − 0.04^*a*^	.59**
		2003–2010	*Y* = 0.14*X* + 0.18*W* − 0.06^*a*^	.73**
	Dis_t	1985–1994	*NS*^*c*^	
		1994–2003	*NS*^*c*^	
		2003–2010	*Y* = 0.03*X* + 0.57*W* + 0.04^*a*^	.45**
	Dis_r	1985–1994	*NS*^*c*^	
		1994–2003	*Y* = −0.03*X* + 0.43*W* + 0.25^*a*^	.61**
		2003–2010	*Y* = 0.02*X* + 0.53*W* + 0.13^*a*^	.54**
ED	AAI	1985–1994	*Y* = 0.01*X* + 0.02*W* − 0.95^*a*^	.44**
		1994–2003	*Y* = 0.02*X* + 0.2*W* + 0.04^*a*^	.67**
		2003–2010	*Y* = 0.01*X* + 0.17*W* + 0.03^*a*^	.73**
	Dis_t	1985–1994	*NS*^*c*^	
		1994–2003	*Y* = 0.02*X* + 0.58*W* + 0.04^*a*^	.51**
		2003–2010	*Y* = 0.01*X* + 0.47*W* + 0.01^*a*^	.52**
	Dis_r	1985–1994	*NS*^*c*^	
		1994–2003	*Y* = 0.01*X* + 0.53*W* + 0.1^*a*^	.58**
		2003–2010	*Y* = −0.06*X* + 0.04*W* + 0.52^*a*^	.60**
AI	AAI	1985–1994	*Y* = −0.01*X* + 0.15*W* + 0.01^*a*^	.57**
		1994–2003	*Y* = −0.14*X* + 0.13*W* + 0.06^*a*^	.72**
		2003–2010	*Y* = −0.01*X* + 0.28*W* + 0.01^*a*^	.68**
	Dis_t	1985–1994	*NS*^*c*^	
		1994–2003	*Y* = 0.02*X* − 0.08^*b*^ (*Lambda* = 0.59)	.57**
		2003–2010	*Y* = −0.12*X* + 0.55*W* − 0.01^*a*^	.49**
	Dis_r	1985–1994	*NS*^*c*^	
		1994–2003	*Y* = 0.07*X* + 0.51*W* − 0.05^*a*^	.63**
		2003–2010	*Y* = 0.04*X* + 0.47*W* − 0.03^*a*^	.55**
SHAPE_AM	AAI	1985–1994	*NS*^*c*^	
		1994–2003	*Y* = −0.03*X* + 0.4*W* + 0.05^*a*^	.36**
		2003–2010	*Y* = −0.06*X* + 0.19*W* + 0.02^*a*^	.52**
	Dis_t	1985–1994	*NS*^*c*^	
		1994–2003	*Y* = 0.04*X* + 0.37*W* + 0.01^*a*^	.32**
		2003–2010	*NS*^*c*^	
	Dis_r	1985–1994	*NS*^*c*^	
		1994–2003	*Y* = −0.01*X* + 0.39*W* + 0.04^*a*^	.31**
		2003–2010	*Y* = −0.08*X* + 0.24*W* − 0.06^*a*^	.45**
SHDI	AAI	1985–1994	*NS*^*c*^	
		1994–2003	*NS*^*c*^	
		2003–2010	*NS*^*c*^	
	Dis_t	1985–1994	*NS*^*c*^	
		1994–2003	*NS*^*c*^	
		2003–2010	*NS*^*c*^	
	Dis_r	1985–1994	*NS*^*c*^	
		1994–2003	*NS*^*c*^	
		2003–2010	*NS*^*c*^	

** Significant at the 99% confidence level.

Abbreviations: patch density (PD), edge density (ED), area-weighted shape index (SHAPE_AM), Shannon’s diversity index (SHDI), aggregation index (AI), anthropogenic activity intensity (AAI), distance to towns (Dis_t), distance to roads (Dis_r).

^*a*^ Spatial lag models; WY = weighted mean of soil landscape metrics for adjacent blocks.

^*b*^ Spatial error models.

^*c*^ No significant relationships were identified by spatial regression.

**Table 5:** Relationships between soil landscape change and rural anthropogenic activity (5*km* scale) obtained by spatial regression.

*Dependent variable* (*Metric*)	*Independent variable* (*Indicator*)	*Time period*	*Spatial regression*	*R*^2^
PD	AAI	1985–1994	*Y* = 1.12*X* – 0.19*W* + 0.01^*a*^	.61**
		1994–2003	*Y* = 1.43*X* + 0.21*W* + 0.03^*a*^	.57**
		2003–2010	*Y* = 1.31*X* + 0.17*W* − 0.03^*a*^	.67**
	Dis_t	1985–1994	*Y* = −0.02*X* − 0.13*W* + 0.05^*a*^	.51**
		1994–2003	*Y* = −0.01*X* + 0.31*W* + 0.33^*a*^	.52**
		2003–2010	*Y* = −0.01*X* + 0.24^*b*^ (*Lambda* = 0.48)	.43**
	Dis_r	1985–1994	*Y* = −0.05*X* − 0.12*W* + 0.05^*a*^	.53**
		1994–2003	*Y* = −0.02*X* + 0.43*W* + 0.19^*a*^	.57**
		2003–2010	*Y* = −0.01*X* + 0.51*W* + 0.08^*a*^	.52**
ED	AAI	1985–1994	*Y* = 0.4*X* + 0.15*W* + 0.03^*a*^	.71**
		1994–2003	*Y* = 0.5*X* + 0.48*W* + 0.03^*a*^	.69**
		2003–2010	*Y* = 0.3*X* + 0.34*W* + 0.04^*a*^	.59**
	Dis_t	1985–1994	*NS*^*c*^	
		1994–2003	*Y* = −0.04*X* + 0.69*W* + 0.03^*a*^	.56**
		2003–2010	*Y* = −0.07*X* + 0.58*W* + 0.01^*a*^	.44**
	Dis_r	1985–1994	*Y* = −0.02*X* + 0.23*W* + 0.15^*a*^	.57**
		1994–2003	*Y* = −0.07*X* + 0.64*W* + 0.06^*a*^	.60**
		2003–2010	*Y* = −0.03*X* + 0.55*W* + 0.02^*a*^	.58**
AI	AAI	1985–1994	*Y* = −0.04*X* + 0.32*W* + 0.03^*a*^	.73**
		1994–2003	*Y* = −0.03*X* + 0.5*W* + 0.08^*a*^	.66**
		2003–2010	*Y* = 0.17*X* + 0.32*W* − 0.16^*a*^	.59**
	Dis_t	1985–1994	*Y* = −0.05*X* + 0.41*W* − 0.02^*a*^	.51**
		1994–2003	*Y* = −0.07*X* + 0.69*W* − 0.01^*a*^	.57**
		2003–2010	*Y* = −0.24*X* + 0.58*W* − 0.08^*a*^	.46**
	Dis_r	1985–1994	*Y* = 0.02*X* + 0.39*W* − 0.01^*a*^	.51**
		1994–2003	*Y* = 0.05*X* + 0.65*W* − 0.03^*a*^	.62**
		2003–2010	*Y* = 0.02*X* + 0.54*W* − 0.01^*a*^	.56**
SHAPE_AM	AAI	1985–1994	*NS*^*c*^	
		1994–2003	*Y* = −0.02*X* + 0.05^*b*^ (*Lambda* = 0.36)	.44**
		2003–2010	*Y* = −0.03*X* + 0.1*W* + 0.12^*a*^	.52**
	Dis_t	1985–1994	*NS*^*c*^	
		1994–2003	*Y* = 0.04*X* + 0.32*W* + 0.06^*a*^	.31**
		2003–2010	*NS*^*c*^	
	Dis_r	1985–1994	*NS*^*c*^	
		1994–2003	*Y* = −0.3*X* + 0.06^*b*^ (*Lambda* = 0.36)	.29**
		2003–2010	*NS*^*c*^	
SHDI	AAI	1985–1994	*Y* = 0.06*X* – 0.02^*b*^ (*Lambda* = 0.21)	.31**
		1994–2003	*Y* = 0.02*X* – 0.02^*b*^ (*Lambda* = 0.26)	.41**
		2003–2010	*NS*^*c*^	
	Dis_t	1985–1994	*NS*^*c*^	
		1994–2003	*Y* = −0.08*X* – 0.02^*b*^ (*Lambda* = 0.26)	.22**
		2003–2010	*NS*^*c*^	
	Dis_r	1985–1994	*NS*^*c*^	
		1994–2003	*Y* = −0.01*X* – 0.02^*b*^ (*Lambda* = 0.27)	.26**
		2003–2010	*NS*^*c*^	

** Significant at the 99% confidence level.

Abbreviations: patch density (PD), edge density (ED), area-weighted shape index (SHAPE_AM), Shannon’s diversity index (SHDI), aggregation index (AI), anthropogenic activity intensity (AAI), distance to towns (Dis_t), distance to roads (Dis_r)

^*a*^ Spatial lag models; WY = weighted mean of soil landscape metrics for adjacent blocks.

^*b*^ Spatial error models.

^*c*^ No significant relationships were identified by spatial regression.

## Discussion

### Impact of anthropogenic activity on soils and soil landscape patterns

As was reported in the present study, 6.7% of soil surface area was lost due to accelerating rural settlement expansion in the Tiaoxi watershed during the period 1985–2010, which constitutes a substantial amount of soil resources in the eastern coastal region of China. Note that many other areas of China also experienced high rates of soil loss due to the built-up land sprawl after the 1980s. Our results implied that with time the soil landscapes were becoming less dominant, more fragmented and more irregular in shape. Besides, the changes were less significant during the time period 2003–2010 than during the period 1994–2003. The AI and SHDI metrics represented a decreasing trend, whereas the PD and ED metrics represented an increasing trend in soil landscape change. Li and Yeh [27] reported that the fast urban expansion has triggered the loss of a large amount of agricultural land in the Pearl River Delta, specifically, a 13.1% of the total agricultural land was lost during 1988–1993 and a 5.8% during 1993–1997. Zhang *et al*. [28] reported that in Nanjing city the percentage of soil area lost to urban use ranged from 4.8% of the area during 1984 to 11.3% of the area during 2003. Similar phenomena were also observed in the Beijing-Tianjin-Hebei region (large urban agglomeration) [29], Su-Xi-Chang region (fast developing costal urban agglomeration) [30] and Yixing city (a fast developing city) [31]. Clearly, this is a serious issue that deserves much more attention by the government than it currently does.

Changes in soil landscape patterns were largely explained by the spatial expansion of built-ups. Sprawled built-ups reduced the integrity of soil landscapes, resulting in a decline in the values of the SHAPE metric as built-ups increased. Anthropogenic activity intensity indicators, like Dis_r and AAI, were proven to be quite useful in the configuration of soil landscape patterns. Roads played a very important role in land development [32], usually influencing the soil landscape patterns (e.g., by blocking soil surface connectivity), which in the present study was represented by a decline in the values of the PD, ED and SHAPE soil metrics. It was found that the AAI indicator had a greater contribution on PD metric changes, implying that in recent years stronger relationships were established between AAI and PD. As regards the AI, SHAPE and SHDI metrics, we found that after 1994 the intensity of anthropogenic activity had a considerable effect on aggregation and diversity, and that after 2003 the intensity of the activity influenced fragmentation more significantly. Regarding the ED metric, our results demonstrated the lack of any trend at the scales considered, suggesting that, in the particular watershed, other anthropogenic activity indicators (such as certain physical or natural factors) may have potentially contributed to the unexpected relationships discussed above.

### Spatiotemporal scale effects

Several previous studies have focused on the description of the response of landscape indicators to scale changes [33, 34, 35], since the spatial pattern is clearly scale-dependent (i.e., it changes with the observation scale). Our research showed that correlations between the intensity of anthropogenic activities and soil landscape patterns varied with landscape block size. Our findings implied that no significant correlations were observed between anthropogenic activities and soil landscape patterns at the 3 km scale, whereas at the 5 km scale all three indicators were significantly correlated with the soil landscape pattern metrics during the period 1994–2003. The R^2^ coefficient of patch density (PD) at the 3 km scale was higher than at the 5 km scale during the time periods 1994–2003 and 2003–2010, demonstrating that the relationship between soil pattern fragmentation and intensity of anthropogenic activity was more significant at the 3 km scale. Similarly, the AI metric was considerably higher at the 3 km scale than at the 5 km scale, suggesting that soil aggregation plays a more significant role at the 3 km scale. However, the SHDI metric had no noticeable correlation with anthropogenic activity intensity variables at the 3 km scale, but exhibited a significant correlation at the 5 km scale, although the R^2^ coefficient was significantly lower (from 0.22 to 0.41) than for other metrics, suggesting that at smaller scales the anthropogenic activity impacts on SHDI were difficult to identify.

It was also found that, not only the spatial scale but also the temporal scale influenced the relationship between soil landscape pattern changes and anthropogenic activity intensity. Other investigators have reported that the temporal scale plays a significant role in analyzing the above relationships [36, 37]. Moreover, Su *et al*. [6] discussed the relationships between rural settlement expansion and paddy soil loss in the Tiaoxi watershed at only one temporal scale (1994–2003). Instead, the present study was more complete, covering three temporal scales (1985–1994, 1994–2003 and 2003–2010). The three different time periods were compared to illustrate the extent of soil landscape pattern changes at different temporal scales, which offered an improved understanding of the relationships between soil landscape pattern change and anthropogenic activity intensity during the 25-year time period. The most serious impacts occurred during the periods 1994–2003 and 2003–2010. Regarding the SHAPE and SHDI metrics, significant correlations were observed during the period 1994–2003 at the 5 km scale. It was concluded that at this scale the SHAPE and SHDI metrics of soil landscape patterns were influenced by significant changes in built-up area expansion over the years. Overall, these findings provide strong evidential support concerning the importance of multi-scale approaches in the determination of the impacts of anthropogenic activity intensity on soil landscape pattern changes.

### Methodological issues

Methodologically, the comprehensive synthesis of spatial analysis, landscape metrics, remote sensing and GIS was proven to be an effective approach in the case of the Tiaoxi watershed. Spatial dependence analysis can help us understand the real effects of incorrect estimation resulting from the implementation of traditional linear regression models that ignore the significant impacts of neighboring sampling sites. As regards the calculation of soil landscape metrics, the spatial lag model was found to be adequate for most landscape metrics at both scales considered (e.g., by revealing relationships accounting for the weighted mean of the dependent variable at adjacent grids). In other words, the spatial patterns of soil landscapes depended not only on the intensity of the local anthropogenic activity, but also on the intensity of activities in neighboring sites.

In addition, at the 5 km scale all models relevant to SHDI were error models, implying that the dependent variable (metric) was affected by a set of observed local indicators, and that the error terms were correlated in a systematic manner across space [38]. As noted earlier, in combination with spatial analysis and landscape metrics, remote sensing images and GIS were valuable tools in the characterization of built-up land by visual interpretation and the delineation of rural settlement expansion. This allowed the systematic mapping of different soil types sealed by anthropogenic activity throughout the Tiaoxi watershed. Lastly, the proposed approach is applicable to other regions experiencing intensive settlement expansion.

### Limitations and implications

The Tiaoxi watershed is a typical one in the developed region of southeastern China [6, 39], which is close to the country’s economic center. Although this work focused on soil landscape pattern changes in response to rural anthropogenic activity, there may exist other factors occasionally linked to soil sealing, such as soil erosion increase, surface water penetration decrease and subsequent increase of surface runoff from sealed surface.

Specifically, in Anji county (within the Tiaoxi watershed) rural anthropogenic activities were associated with soil erosion [39] that controls the discharge of nitrogen and phosphorus from agricultural fields and build-up areas. Eutrophication in rivers and lakes has been a concern across the country during the past decade [39, 40]. In Taihu lake, a severe algal bloom occurred on May 28, 2007, cutting off the tap water supply of about four million local residents for more than four days (we notice that the Tiaoxi watershed accounts for about 70% of the natural freshwater flowing into the Taihu lake annually). Thereafter, a mega research/engineering project was initiated in 2008 with the goal to control eutrophication in the Tiaoxi watershed [39]. Similarly, huge capitals have been invested on pollution control in many other fresh water systems across the country. Non-point source pollution from agricultural fields and domestic sewage from rural areas are the main contributors of nitrogen and phosphorous, the two dominant nutrients causing eutrophication [39, 41].

The surface water penetration decrease and the subsequent surface runoff increase in sealed areas have led to an elevated risk of surface waste washing into water systems. In the Qiantang watershed, next to the Tiaoxi watershed, built-up land was one of the primary predictors of certain hazardous chemical contamination patterns, whereas land use/cover types were good predictors of cyanide and heavy metal changes in river systems [40, 41]. In addition, although water logging caused by rainstorm in both urban and rural areas rarely occurred before, in recent years it has become a common hazard that occurs almost yearly. Obviously, surface sealing in urban and rural areas is one of the main causes.

Rural anthropogenic activities have also affected ecosystem function changes [42, 43, 44]. Long-term monitoring, comprehensive observations and further in-depth analysis are urgently needed to quantify systematically the relation between anthropogenic activity and soil erosion, surface runoff, ecosystem changes etc. Lastly, it should be mentioned that the obtained relationships are case-specific and they do not necessarily denote causation. More observation should be carried to prove the presence of causation.

## Conclusions

As regards important issues of environmental change, rural anthropogenic activity was found to pose significant impacts on soil resources. The main findings of this work, briefly, were as follows: (1) as a result of rural anthropogenic activity, the soil landscape exhibited clear pattern changes, which became more fragmented, more isolated, and less diverse during the last 25 years in the Tiaoxi watershed; (2) 6.7% of the soil was lost under accelerated rural settlement expansion in the Tiaoxi watershed during the period 1985–2010, whereas the observed relationships between anthropogenic activity indicators and soil landscape patterns showed that the increased intensity of anthropogenic activity led to irregular soil shape and edges; (3) correlations between anthropogenic activity intensity and soil landscape patterns varied with landscape block size and temporal scale; (4) multiple-scale approaches played a crucial role in the assessment of anthropogenic activity impacts on soil landscape pattern changes; (5) the comprehensive synthesis of spatial analysis, landscape metrics, remote sensing and GIS proved to be an effective approach in the study of relationships between indicators of anthropogenic activity and changes of soil landscape patterns (an approach that can be applicable to other regions experiencing intensive settlement expansion). Concluding, the findings of the present work suggested that the soil sealing issue deserves much more attention by the government and the public than it currently does.
